# Effectiveness of a Brief Self-determination Theory–Based Smoking Cessation Intervention for Smokers at Emergency Departments in Hong Kong: A Randomized Clinical Trial

**DOI:** 10.1001/jamainternmed.2019.5176

**Published:** 2019-12-02

**Authors:** William Ho Cheung Li, Ka Yan Ho, Man Ping Wang, Derek Yee Tak Cheung, Katherine Ka Wai Lam, Wei Xia, Kai Yeung Cheung, Carlos King Ho Wong, Sophia Siu Chee Chan, Tai Hing Lam

**Affiliations:** 1 School of Nursing, The University of Hong Kong, Hong Kong, China; 2 School of Nursing, Hong Kong Polytechnic University, Hong Kong, China; 3 United Christian Hospital, Hospital Authority, Hong Kong, China; 4 Department of Family Medicine and Primary Care, The University of Hong Kong, Hong Kong, China; 5 School of Public Health, The University of Hong Kong, Hong Kong, China

## Abstract

**Importance:**

Clinicians have an opportunity to provide smoking cessation interventions to smokers who present to emergency departments (EDs). The effectiveness of a brief intervention based on self-determination theory for smoking cessation is uncertain.

**Objective:**

To examine the effectiveness of a brief intervention based on self-determination theory for smoking cessation (immediate or progressive) among Chinese smokers presenting at EDs in Hong Kong.

**Design, Setting, and Participants:**

This single-blind, multicenter intent-to-treat randomized clinical trial was conducted at the EDs of 4 major acute care hospitals in different districts of Hong Kong. In total, 1571 smokers 18 years or older who presented at 4 major EDs between July 4, 2015, and March 17, 2017, were randomized into an intervention group (n = 787) and a control group (n = 784).

**Interventions:**

The intervention group received brief advice (about 1 minute) and could choose their own quit schedules (immediate or progressive). The control group received a smoking cessation leaflet.

**Main Outcomes and Measures:**

Follow-up visits were conducted at 1, 3, 6, and 12 months. The primary outcome measure, by intent to treat, was biochemically validated abstinence at 6 months.

**Results:**

Participants (N = 1571) included 1381 men (87.9%); the mean (SD) age at baseline was 47.4 (16.4) years. Among participants who self-reported abstinence at 6 months, 50.3% (85 of 169) had biochemical validation by both an exhaled carbon monoxide test and a saliva cotinine test. Compared with the control group, the intervention group had statistically higher biochemically validated abstinence at 6 months: 6.7% (53 of 787) vs 2.8% (22 of 784) (*P* < .001), with an adjusted relative risk of 3.21 (95% CI, 1.74-5.93; *P* < .001). The intervention group also had higher self-reported quit rates at 6 months (12.2% [96 of 787] vs 9.3% [73 of 784], *P* = .04) and 12 months (13.0% [102 of 787] vs 8.5% [67 of 784], *P* < .01), as well as higher biochemically validated abstinence at 12 months (7.0% [55 of 787] vs 3.7% [29 of 784], *P* < .001). The additional cost for each intervention group participant was US $0.47, with an estimated gain of 0.0238 quality-adjusted life-year. The incremental cost per quality-adjusted life-year (US $19.53) fell within acceptable thresholds.

**Conclusions and Relevance:**

This brief, low-cost self-determination theory–based intervention for smokers presenting at EDs effectively increased the biochemically validated quit rate at 6 months. If delivered routinely, such a simple intervention may offer a cost-effective and sustainable approach to help many smokers quit smoking.

**Trial Registration:**

ClinicalTrials.gov identifier: NCT02660957

## Key Points

**Question:**

What is the effect of a brief intervention based on self-determination theory compared with a smoking cessation leaflet on promoting abstinence in smokers presenting at emergency departments?

**Findings:**

In this randomized clinical trial of 1571 smokers who presented at emergency departments, a self-determination theory–based intervention was effective in increasing the biochemically validated quit rate at 6 months compared with a smoking cessation leaflet (53 of 787 [6.7%] vs 22 of 784 [2.8%]).

**Meaning:**

The findings suggest that if delivered routinely, this brief self-determination theory–based intervention offers a cost-effective and sustainable approach to help smokers quit smoking.

## Teaser

This randomized clinical trial examines the effectiveness of a brief intervention based on self-determination theory for smoking cessation among Chinese smokers presenting at emergency departments in Hong Kong.

## Introduction

Presentation at emergency departments (EDs) represent an excellent teachable moment for smoking cessation interventions. A 2013 systematic review and meta-analysis^1^ of randomized clinical trials (RCTs) on the efficacy of smoking cessation interventions in EDs identified 7 trials. Two trials reported significant intervention benefit at the 1-month follow-up, but no trials reported significant differences between study groups at 3, 6, and 12 months. Moreover, none of the reviewed trials were rated as methodologically strong, and most were underpowered; 4 studies randomized fewer than 100 participants. In addition, all of the above-mentioned trials were conducted in Western countries.

Previous smoking cessation trials^2,3,4^ in outpatient clinics in Hong Kong revealed that many smokers were reluctant to quit but were interested in reducing the number of cigarettes smoked per day. Therefore, a potential strategy to support smoking cessation is allowing smokers to choose if they want to quit immediately or quit progressively, with the ultimate goal of complete cessation.

The present intervention was guided by self-determination theory,^5^ which states that behavioral regulation is more autonomous when it is internalized, rather than being regulated by external factors (eg, requests from family members, friends, or health care professionals). Compared with external regulation, autonomous regulation is associated with increased self-efficacy, greater behavioral persistence, long-term behavioral changes, and more positive health behavior.^6^ Autonomy is a behavioral factor that is emphasized by freedom of choice.^5^ Previous RCTs^7,8,9^ have reported that patients who had the opportunity to decide on their own treatment would be more ready to comply with instructions. There is some evidence that supports a positive association between autonomy and competence,^8,9^ with people who have greater autonomy demonstrating greater competence in achieving behavioral change compared with those with less autonomy. Therefore, we anticipated that smokers’ autonomy and competence would increase if they experienced volition and choice in their behavior, thereby enhancing their self-efficacy to quit smoking. The present RCT tested the effectiveness of a brief, low-cost self-determination theory–based smoking cessation (immediate or progressive) intervention among Chinese smokers who presented at EDs in Hong Kong.

## Methods

### Study Design

A single-blind, multicenter RCT was conducted at the EDs in 4 major acute-care hospitals in different districts of Hong Kong. This study followed the Consolidated Standards of Reporting Trials (CONSORT) 2010 guideline. Ethical approval was obtained from the Institutional Review Board of the University of Hong Kong/Hospital Authority Hong Kong West Cluster (UW 14-528). The trial protocol is available in Supplement 1. Participants provided written informed consent.

### Study Sample and Recruitment

In Hong Kong EDs, patients are prioritized for treatment by a triage system with the following 5 different levels: critical (level 1), emergency (level 2), urgent (level 3), semiurgent (level 4), and nonurgent (level 5).^10^ Approximately 68% of patients are triaged as semiurgent or nonurgent^11^ and generally need to wait for more than 30 minutes, with some waiting for 1 to 3 hours.^12^ This long waiting time offers an opportunity for health care professionals to advise smokers about quitting and provide information about available smoking cessation services.

Chinese smokers who presented at the selected EDs and fulfilled the inclusion criteria were invited to participate in this RCT (Figure). The inclusion criteria were (1) age 18 years or older, (2) triage as semiurgent (level 4) or nonurgent (level 5), and (3) current smokers (occasional or daily). Smokers were excluded if they were unable to give informed written consent or receive counseling because of impaired mental status, cognitive impairment, or communication barriers or if they had participated in other smoking cessation programs or services. All smokers were first referred after triage by triage nurses in accordance with the Hospital Authority guidelines. Eligible participants were then approached by the research assistants.

**Figure.**
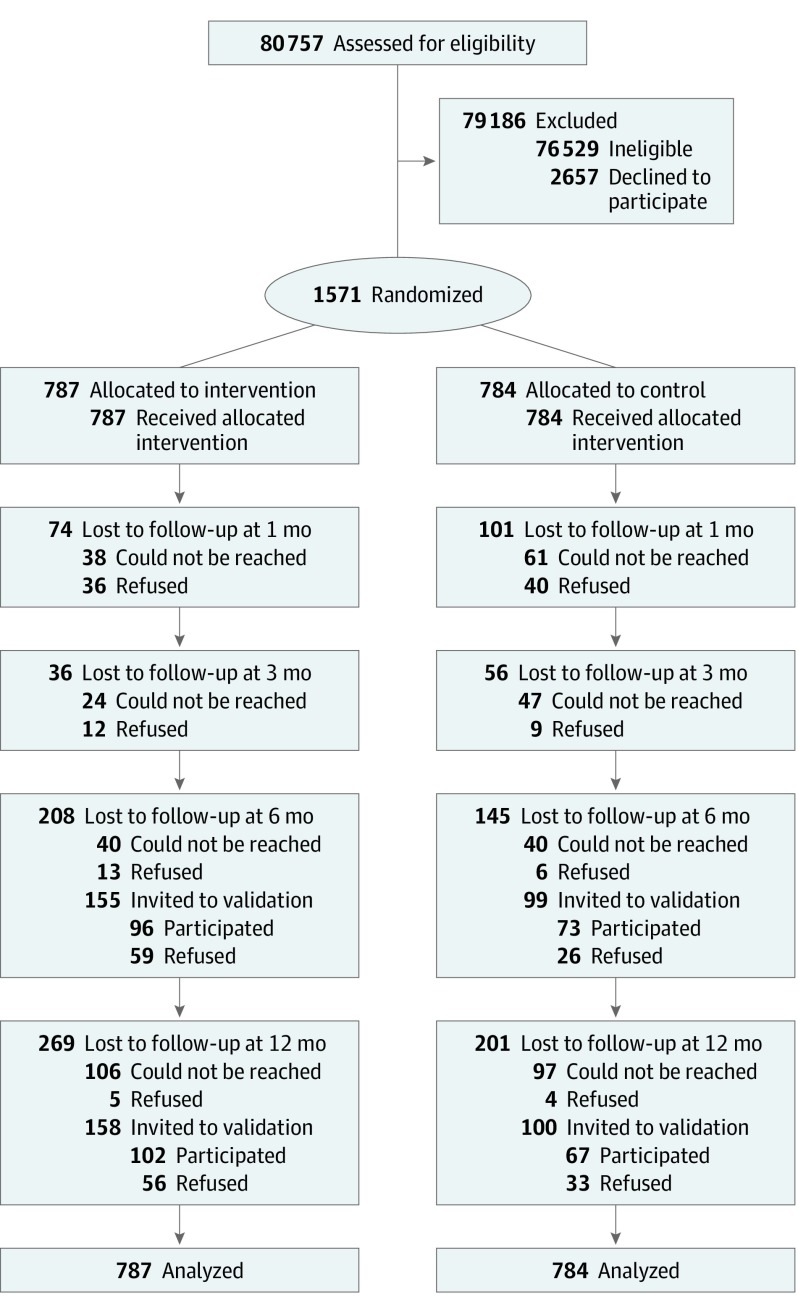
CONSORT Flow Diagram

### Randomization

Participants were randomized to either the control group or the intervention group. Randomization was performed by a research assistant, who opened a serially labeled, opaque, and sealed envelope with a card inside indicating the group allocation. The random numbers used for allocation were generated before participant recruitment by another research assistant using a personal computer. The baseline assessment and intervention for each participant were implemented in a single room or cubicle. This ensured participants’ privacy and prevented the possibility of interaction between participants in the 2 groups in the same setting while waiting for medical consultation.

### Sample Size Calculation

The sample size was estimated according to a previous RCT^13^ of a smoking reduction plus nicotine replacement therapy intervention involving 1154 Chinese adult smokers unwilling to quit smoking (biochemically validated quit rate of 4.4% [10 of 226] in the control group and 8.0% [74 of 928] in the intervention group at 6 months). To detect a 2-sided significant difference between groups with a power of 80% and significance level of 5%, which is commonly accepted in behavioral studies,^14^ the required sample size was estimated to be 1088 participants (544 in each group). Given an expected retention rate of approximately 70% at the 6-month follow-up, the target was at least 1554 individuals (777 in each group).

### Interventions

The intervention group received brief advice provided by trained retired nurses using the AWARD model, which includes the following steps: (1) ask about smoking history, (2) warn about the high mortality risk that 1 in 2 smokers will be killed by smoking,^15^ (3) advise to quit now, (4) refer smokers to smoking cessation hotline services, and (5) do it again. The AWARD model was validated in previous clinical trials on smoking cessation.^3,8,16^ In addition, participants could choose their own quit schedules (immediate or progressive). The control group received a smoking cessation leaflet and placebo treatment. Four 1-minute simple booster calls and placebo booster calls were also provided to the intervention and control groups, respectively. Additional descriptions of interventions are provided in the eAppendix in Supplement 2.

### Measures

#### Baseline Measures

Participants’ baseline data, including demographic characteristics, health status, and smoking history, were obtained using a structured questionnaire based on previous trials.^17,18^ The questionnaires were administered face-to-face by a trained research assistant before randomization.

#### Primary Outcome Measure

Follow-up visits were conducted at 1, 3, 6, and 12 months. The primary outcome measure was biochemically validated abstinence at 6 months. This was originally a secondary outcome; however, before the start of recruitment, the reviewer from the grant review board (masked review, written communication, October 2014) suggested that we change it to the primary outcome measure.

#### Secondary Outcomes

The secondary outcomes included (1) biochemically validated abstinence at 12 months, (2) self-reported 7-day point prevalence of abstinence at 6 and 12 months, (3) self-reported reduction of at least 50% in daily cigarette consumption at 6 and 12 months, (4) self-efficacy against tobacco by the 12-item Smoking Self-Efficacy Questionnaire (SEQ-12) Chinese version^19^ at 6 and 12 months, and (5) the health utility score by the Short-Form Six-Dimension (SF-6D) Chinese version^20^ at 6 and 12 months. Individuals with self-reported smoking abstinence for more than 7 days were invited to participate in a biochemical validation test. The biochemically validated 7-day point prevalence of abstinence was confirmed by a carbon monoxide level in expired air of less than 4 ppm and by a saliva cotinine level of less than 115 ng/mL in a parallel test,^21^ which provided good agreement with self-reported smoking status.^22,23^ Only the individuals who passed both of the validation tests were regarded as having biochemically validated abstinence; otherwise, participants were considered to have failed the validation.

#### Instruments

A structured standardized questionnaire was developed by adopting and modifying international and locally validated instruments from previous trials.^17,18^ Participants’ self-efficacy against tobacco was assessed by the SEQ-12, which is a valid and reliable scale that has been used in both research and clinical settings with Chinese populations in Hong Kong.^19^ The health utility score was measured with the SF-6D, which reflects the level of physical, mental, and social functioning associated with a particular health state, as well as the preference weight that the general population gives to that health state.^20^ Utility scores for individual health states are combined with the survival time in each health state to calculate quality-adjusted life-years (QALYs), a health outcome measure that combines quality of life with length of life. The Panel on Cost-Effectiveness in Health and Medicine of the US Public Health Service recommends the use of QALYs for cost-effectiveness analyses of health-related interventions.^24^ The Chinese version of the SF-6D and its scoring algorithm have been validated and are applicable for the Chinese population in Hong Kong.^20^

### Statistical Analysis

Data analysis was performed using IBM SPSS Statistics for Windows (version 23.0; IBM). Baseline characteristics of participants in the intervention and control groups were first compared using χ^2^ tests for categorical variables and independent sample *t* tests for continuous variables. The primary analysis was the unadjusted difference in biochemically validated quit rates at 6 months between the intervention and control groups. A 2-tailed Fisher exact test was used if there were 5 or fewer participants per cell. A similar approach was used to analyze the differences in secondary outcomes. The number needed to treat, which indicates the number of treated individuals needed for 1 additional successful outcome, was computed using the reciprocal of the quit rate difference between the intervention and control groups.^25^ A generalized estimating equation (GEE) model was used to calculate adjusted relative risks (aRRs) for validated and self-reported abstinence after adjusting for the clustering effect (EDs), any baseline demographic or clinical characteristics that showed significant differences, and variables that have been reported to be associated with smoking cessation.^26^ The GEE model was built (based on the goodness of fit) after checking for multicollinearity among the identified variables. The model that had the lowest quasi-Akaike information criterion was selected as the best model. By intent-to-treat analysis, participants who were lost to follow-up or refused to participate in the validation tests were considered smokers with no reduction in cigarette consumption compared with baseline. Multiple imputation was used to handle missing values for demographic information at baseline.^27^ Sensitivity analyses for the abstinence outcomes were performed for multiple imputations and by complete cases to assess differences in the results.

The cost of each intervention was recorded, including direct operating expenses (eg, salary of staff members and materials used for the training of counselors), participant recruitment, and intervention delivery (eg, boosters). The cost per person of providing the brief advice using the AWARD model was calculated by dividing the total cost by the number of smokers in each group. The SF-6D health utility scores at baseline and at 6 and 12 months were used to construct 1-year QALYs using the standard area under the receiver operating characteristic curve. Total QALYs were estimated for the intervention and control groups. We reported the incremental cost-effectiveness ratio (ICER) in terms of incremental cost per QALY saved by the intervention as follows: (cost of intervention − cost of control) / (QALY of intervention − QALY of control).^28^ The intervention was regarded as cost-effective over control if the ICER was less than 1 to 3 times the gross domestic product per capita, as indicated by the World Health Organization^29^ and cost-effectiveness threshold (US $18 609) derived by opportunity cost in Hong Kong.^30^

## Results

Between July 4, 2015, and March 17, 2017, a total of 1571 smokers who presented at 4 major EDs consented to participate in this RCT and were randomized into an intervention group (n = 787) and a control group (n = 784). Participants included 1381 men and 190 women with a mean (SD) age at baseline of 47.4 (16.4) years (Table 1). Individuals in the intervention and control groups had similar demographic characteristics and smoking profiles. The retention rates at 6 and 12 months were 68.5% (1076 of 1571) and 64.2% (1009 of 1571), respectively. Among participants who self-reported abstinence at 6 and at 12 months, biochemical validation by findings from both an exhaled carbon monoxide test and a saliva cotinine test was performed for 50.3% (85 of 169) at 6 months and for 52.7% (89 of 169) at 12 months; validation was performed for 75 of 85 participants (88.2%) at 6 months and for 84 of 89 participants (94.4%) at 12 months. The Figure shows the CONSORT flowchart.

**Table 1.** Participants’ Baseline Demographic Characteristics and Smoking Profiles

Variable	No./Total No. (%)^a^		*P* Value
	Intervention (n = 787)	Control (n = 784)	
Age, mean (SD), y	46.5 (16.0)	48.0 (16.8)	.08
Sex			
Male	685/784 (87.4)	696/780 (89.2)	.25
Female	99/784 (12.6)	84/780 (10.8)	
Marital status			
Single	228/771 (29.6)	193/762 (25.3)	.04
Married or cohabiting	453/771 (58.8)	495/762 (65.0)	
Divorced, separated, or widowed	90/771 (11.7)	74/762 (9.7)	
Employment status			
Unemployed or retired	188/775 (24.3)	202/762 (26.5)	.33
Employed	587/775 (75.7)	560/762 (73.5)	
Educational level			
Primary or below	190/769 (24.7)	205/760 (27.0)	.38
Secondary	531/769 (69.1)	500/760 (65.8)	
Tertiary	48/769 (6.2)	55/760 (7.2)	
Monthly household income, US $			
<1275	208/662 (31.4)	226/652 (34.7)	.10
1275-3825	369/662 (55.7)	326/652 (50.0)	
≥3825	85/662 (12.8)	100/652 (15.3)	
Smoking-related chronic disease			
Yes	162/787 (20.6)	163/784 (20.8)	.92
No	625/787 (79.4)	621/784 (79.2)	
Health utility score by the SF-6D, mean (SD)^b^	0.58 (0.20)	0.57 (0.19)	.58
Daily cigarette consumption, mean (SD), No.^c^	14.0 (7.9)	13.6 (7.6)	.14
Nicotine dependence by the Heaviness of Smoking Index^c^^,^^d^			
Light, ≤2	388/776 (50.0)	391/774 (50.5)	.84
Moderate to heavy, 3-6	388/776 (50.0)	383/774 (49.5)	
Age at starting smoking weekly, mean (SD), y	17.3 (5.8)	17.6 (6.5)	.10
Tried to quit smoking for >24 h^c^			
No	235/785 (29.9)	265/781 (33.9)	.05
Yes	550/785 (70.1)	516/781 (66.1)	
Tried to reduce smoking for >24 h^c^			
No	387/778 (49.7)	400/774 (51.7)	.24
Yes	391/778 (50.3)	374/774 (48.3)	
Readiness to quit			
≤30 d	203/677 (30.0)	218/775 (28.1)	.44
>30 d	474/677 (70.0)	557/775 (71.9)	
Self-efficacy against tobacco by the SEQ-12, mean (SD)^e^	28.80 (11.06)	28.16 (11.10)	.35

Abbreviations: SEQ-12, Smoking Self-Efficacy Questionnaire; SF-6D, Short-Form Six-Dimension.

^a^ Sample sizes varied because of missing data on some variables.

^b^ The SF-6D is composed of 6 multilevel dimensions. The SF-6D scores were weighted from a sample of the general population, which ranged from 0 to 1.

^c^ There were statistically significant differences between individuals who chose to quit immediately and those who chose to quit progressively (*P* < .001 for all).

^d^ The Heaviness of Smoking Index,^31^ a 2-item index from multiple-choice response options (0-3), was determined by assessing cigarettes smoked per day and latency to smoke after waking; the higher the indexes, the greater smoking nicotine dependence.

^e^ On a 12-item 5-point Likert-type scale in the SEQ-12, responses ranged from “not at all sure” to “absolutely sure.” A summary score of the SEQ-12 ranged from 12 to 60, with higher scores indicating higher self-efficacy.

The primary outcome measure (biochemically validated abstinence rate) was statistically significantly higher in the intervention group than the control group at 6 months (6.7% [53 of 787] vs 2.8% [22 of 784], *P* < .001), with a 95% CI of 2.0% to 6.0% for the 3.9% difference between the 2 population proportions (Table 2). The number needed to treat for the intervention was 25.6.

**Table 2.** Smoking Cessation Outcomes of Participants in the Intervention and Control Groups

Variable	6 mo		*P* Value	12 mo		*P* Value
	Intervention Group (n = 787)	Control Group (n = 784)		Intervention Group (n = 787)	Control Group (n = 784)	
Biochemically validated abstinence primary outcome measure, No. (%)	53 (6.7)	22 (2.8)	<.001	55 (7.0)	29 (3.7)	.001
Self-reported 7-d point prevalence of abstinence, No. (%)	96 (12.2)	73 (9.3)	.04	102 (13.0)	67 (8.5)	.005
Self-reported reduction of ≥50% in daily cigarette consumption, No./total No. (%)^a^	123/691 (17.8)	127/711 (17.9)	.98	130/685 (19.0)	105/717 (14.6)	.03
Self-reported quitter or reduction of ≥50% in daily cigarette consumption, No. (%)	219 (27.8)	200 (25.5)	.16	232 (29.5)	172 (21.9)	<.001
Self-efficacy against tobacco by the SEQ-12, mean (SD)^b^	37.88 (11.70)	36.95 (11.30)	.11	35.49 (10.33)	34.27 (9.97)	.02
Health utility score by the SF-6D, mean (SD)^c^	0.71 (0.10)	0.67 (0.10)	<.001	0.70 (0.13)	0.67 (0.14)	<.001

Abbreviations: SEQ-12, Smoking Self-Efficacy Questionnaire; SF-6D, Short-Form Six-Dimension (SF-6D).

^a^ By intent-to-treat analysis, assuming all nonrespondents were current smokers, did not make a quit attempt, and did not change their smoking behavior during the follow-up period compared with baseline.

^b^ On a 12-item 5-point Likert-type scale in the SEQ-12, responses ranged from “not at all sure” to “absolutely sure.” A summary score of the SEQ-12 ranged from 12 to 60, with higher scores indicating higher self-efficacy.

^c^ The SF-6D is composed of 6 multilevel dimensions. The SF-6D scores were weighted from a sample of the general population, which ranged from 0 to 1.

Compared with the control group, the intervention group also had statistically significantly higher self-reported 7-day point prevalence of abstinence at 6 months (12.2% [96 of 787] vs 9.3% [73 of 784], *P* = .04) and 12 months (13.0% [102 of 787] vs 8.5% [67 of 784], *P* < .01) and had statistically significantly higher biochemically validated abstinence at 12 months (7.0% [55 of 787] vs 3.7% [29 of 784], *P* < .001). Excluding quitters, self-reported reduction of at least 50% in daily cigarette consumption was similar in the 2 groups (17.8% [123 of 691] vs 17.9% [127 of 711], *P* = .98) at the 6-month follow-up but was statistically significantly higher in the intervention group at 12 months (20.0% [130 of 685] vs 14.6% [105 of 717], *P* = .03). Of all the participants, 27 requested referral to a smoking cessation service, while only 7 of them had validated abstinence.

Self-efficacy against tobacco at 12 months measured by the SEQ-12 (35.49 vs 34.27, *P* = .02) and the SF-6D health utility score at 6 months (0.71 vs 0.67, *P* < .001) and 12 months (0.70 vs 0.67, *P* < .01) were statistically significantly higher in the intervention group than in the control group. The intervention saved 0.0238 QALY (0.6782 vs 0.6544, *P* = .002) over the 1-year trial period.

By intent to treat, the GEE model revealed that the intervention group had statistically significantly higher biochemically validated abstinence than the control group at both 6 months (aRR, 3.21; 95% CI, 1.74-5.93; *P* < .001) and 12 months (aRR, 2.23; 95% CI, 1.25-3.97; *P* = .004) after adjusting for age, sex, marital status, employment status, educational level, monthly household income, smoking-related chronic disease, quality of life, self-efficacy against tobacco by the SEQ-12 at baseline, and nicotine dependence (Table 3). Although self-reported abstinence was higher in the intervention group than in the control group at 6 months, the difference was not statistically significant (aRR, 1.32; 95% CI, 0.90-1.95; *P* = .11) after controlling for the previously mentioned variables. However, the difference was statistically significant at 12 months (aRR, 1.46; 95% CI, 1.06-2.19; *P* = .04) (Table 4). The sensitivity analyses for the use of multiple imputations and by complete cases yielded results similar to those of the main analyses (eTable in Supplement 2).

**Table 3.** Generalized Estimating Equation for Biochemically Validated Abstinence at 6- and 12-Month Follow-up Visits Among 1571 Participants^a^

Variable	6 mo		12 mo	
	aRR (95% CI)	*P* Value	aRR (95% CI)	*P* Value
Study group				
Intervention	3.21 (1.74-5.93)	<.001	2.23 (1.25-3.97)	.004
Control	1 [Reference]	NA	1 [Reference]	NA
Age	1.01 (0.98-1.05)	.74	1.00 (0.99-1.02)	.59
Sex				
Male	0.36 (0.15-0.84)	.02	0.92 (0.32-2.62)	.87
Female	1 [Reference]	NA	1 [Reference]	NA
Marital status				
Single	0.61 (0.27-1.34)	.20	0.54 (0.25-1.19)	.11
Divorced, separated, or widowed	0.73 (0.26-2.04)	.54	0.73 (0.28-1.88)	.50
Married or cohabiting	1 [Reference]	NA	1 [Reference]	NA
Employment status				
Unemployed or retired	1.24 (0.49-3.17)	.64	1.37 (0.66-2.83)	.38
Employed	1 [Reference]	NA	1 [Reference]	NA
Educational level				
Primary or below	0.57 (0.17-1.84)	.33	0.93 (0.26-3.37)	.92
Secondary	0.61 (0.23-1.58)	.29	0.65 (0.23-1.80)	.39
Tertiary	1 [Reference]	NA	1 [Reference]	NA
Monthly household income, US $				
<1275	0.72 (0.23-2.23)	.56	1.08 (0.40-2.91)	.88
1275-3825	1.02 (0.44-2.37)	.96	1.40 (0.57-3.40)	.44
≥3825	1 [Reference]	NA	1 [Reference]	NA
Smoking-related chronic disease				
Yes	1.15 (0.58-2.25)	.88	1.20 (0.62-2.34)	.57
No	1 [Reference]	NA	1 [Reference]	NA
Health utility score by the SF-6D^b^	0.19 (0.01-3.68)	.27	0.08 (0.01-1.69)	.09
Self-efficacy against tobacco by the SEQ-12^c^	1.02 (0.97-1.07)	.11	1.02 (0.99-1.05)	.13
Nicotine dependence by the Heaviness of Smoking Index^d^				
Moderate to heavy	0.31 (0.15-0.61)	.001	0.38 (0.20-0.72)	.002
Light	1 [Reference]	NA	1 [Reference]	NA

Abbreviations: aRR, adjusted relative risk; NA, not applicable; SEQ-12, Smoking Self-Efficacy Questionnaire; SF-6D, Short-Form Six-Dimension.

^a^ The generalized estimating equation model was built to derive RRs based on the goodness of fit. The variables that were statistically significant in the logistic regression and that have been shown to be associated with smoking cessation were selected in the model, and then other variables were put in the model 1 by 1. The model with the least quasi-Akaike information criterion was selected as the best model.

^b^ The SF-6D is composed of 6 multilevel dimensions. The SF-6D scores were weighted from a sample of the general population, which ranged from 0 to 1.

^c^ On a 12-item 5-point Likert-type scale in the SEQ-12, responses ranged from “not at all sure” to “absolutely sure.” A summary score of the SEQ-12 ranged from 12 to 60, with higher scores indicating higher self-efficacy.

^d^ The Heaviness of Smoking Index,^31^ a 2-item index from multiple-choice response options (0-3), was determined by assessing cigarettes smoked per day and latency to smoke after waking; the higher the indexes, the greater the smoking nicotine dependence.

**Table 4.** Generalized Estimating Equation for Self-reported Abstinence at 6- and 12-Month Follow-up Visits Among 1571 Participants^a^

Variable	6 mo		12 mo	
	aRR (95% CI)	*P* Value	aRR (95% CI)	*P* Value
Study group				
Intervention	1.32 (0.90-1.95)	.11	1.46 (1.06-2.19)	.04
Control	1 [Reference]	NA	1 [Reference]	NA
Age	1.00 (0.98-1.02)	.99	1.00 (0.98-1.02)	.98
Sex				
Male	0.43 (0.25-0.70)	.001	0.65 (0.38-1.23)	.15
Female	1 [Reference]	NA	1 [Reference]	NA
Marital status				
Single	0.65 (0.38-1.12)	.10	0.72 (0.42-1.22)	.18
Divorced, separated, or widowed	0.73 (0.38-1.39)	.29	0.31 (0.13-0.73)	.005
Married or cohabiting	1 [Reference]	NA	1 [Reference]	NA
Employment status				
Unemployed or retired	1.13 (0.65-1.94)	.74	1.26 (0.74-2.14)	.35
Employed	1 [Reference]	NA	1 [Reference]	NA
Educational level				
Primary or below	0.64 (0.29-1.43)	.25	0.60 (0.26-1.41)	.22
Secondary	0.71 (0.37-1.38)	.28	0.61 (0.30-1.24)	.14
Tertiary	1 [Reference]	NA	1 [Reference]	NA
Monthly household income, US $				
<1275	0.80 (0.38-1.71)	.53	0.90 (0.45-1.81)	.74
1275-3825	0.92 (0.53-1.57)	.72	0.87 (0.49-1.53)	.61
≥3825	1 [Reference]	NA	1 [Reference]	NA
Smoking-related chronic disease				
Yes	1.03 (0.62-1.71)	.91	1.37 (0.86-2.18)	.14
No	1 [Reference]	NA	1 [Reference]	NA
Health utility score by the SF-6D^b^	0.10 (0.01-0.79)	.02	0.21 (0.03-1.52)	.12
Self-efficacy against tobacco by the SEQ-12^c^	1.02 (1.00-1.03)	.01	1.02 (1.00-1.04)	.03
Nicotine dependence by the Heaviness of Smoking Index^d^				
Moderate to heavy	0.52 (0.35-0.79)	.001	0.58 (0.38-0.88)	.007
Light	1 [Reference]	NA	1 [Reference]	NA

Abbreviations: aRR, adjusted relative risk; NA, not applicable; SEQ-12, Smoking Self-Efficacy Questionnaire; SF-6D, Short-Form Six-Dimension.

^a^ The generalized estimating equation model was built to derive relative risks based on the goodness of fit. The variables that were statistically significant in the logistic regression and that have been shown to be associated with smoking cessation were selected in the model, then other variables were put in the model one by one. The model with the least quasi-Akaike information criterion was selected as the best model.

^b^ The SF-6D is composed of 6 multilevel dimensions. The SF-6D scores were weighted from a sample of the general population, which ranged from 0 to 1.

^c^ On a 12-item 5-point Likert-type scale in the SEQ-12, responses ranged from “not at all sure” to “absolutely sure.” A summary score of the SEQ-12 ranged from 12 to 60, with higher scores indicating higher self-efficacy.

^d^ The Heaviness of Smoking Index,^31^ a 2-item index from multiple-choice response options (0-3), was determined by assessing cigarettes smoked per day and latency to smoke after waking; the higher the indexes, the greater smoking nicotine dependence.

Regarding cost and cost-effectiveness, the additional cost for each intervention group participant was US $0.47, with an estimated 0.0238 QALY gained and an ICER of US $19.53. The ICER value was within the acceptable threshold, which is defined as social willingness to pay for health benefits (US $191 according to a previous study^32^). Additional details regarding the intervention costs are provided in the eAppendix in Supplement 2.

## Discussion

This RCT found that a self-determination theory–based smoking cessation intervention may help smokers presenting at EDs quit smoking. The intervention provided brief advice that highlighted warnings regarding the mortality risk associated with smoking. The results revealed that the intervention was effective in doubling the biochemically validated quit rate at 6 months compared with a smoking cessation leaflet and promoting other smoking cessation outcomes. The effectiveness may be attributable to the fact that our intervention allowed participants in the intervention group to choose their own quitting schedules (immediate or progressive), with the ultimate goal of complete cessation after discussion with the counselor.

Most existing cessation programs, including stage-matched interventions and motivational interviewing, generally require implementation time exceeding 30 minutes, which is often not feasible in busy clinical settings. Previous smoking cessation RCTs^17,18,33^ in outpatient clinics revealed that many patients were unwilling to undergo a long intervention by nonphysicians, and some were reluctant to participate for fear of missing or delaying their medical consultation. Moreover, because of the busy clinical environment and lack of training on smoking cessation, many health care professionals were hesitant to help smokers quit. The present intervention based on the AWARD model was brief (approximately 1 minute), low cost, and simple, but it included strong warning messages about smoking (the high absolute risk for death).^16^ This intervention may be cost-effective and feasible for routine use in clinical practice by most health care professionals or volunteers after minimal training.

Smoking cessation services in Hong Kong provided by government and nongovernmental organizations^34^ have low use rates, with only 23.2% of smokers having tried such services.^35^ The low use may be attributable to the passive approach of these services in Hong Kong (and elsewhere) because smokers must take the initiative to seek help. The present trial adopted an outreach approach to proactively recruit smokers presenting at EDs and help them quit smoking. The present study’s findings may be used to create a new smoking cessation service model using a brief, flexible, low-cost, and proactive approach to help smokers who present at EDs quit smoking.

### Implications for Clinical Practice

This innovative, low-cost, effective, and brief self-determination theory–based intervention may represent an important contribution to clinical practice regarding brief smoking cessation. Our results support the development of new evidence-based proactive smoking cessation services. Strategies to support such services include hiring and training retired and existing nurses and other health care workers in EDs. In addition, clinical practice guidelines are needed that can motivate more health care workers to routinely promote smoking cessation to smokers in clinical and community settings beyond EDs. Although intervention group participants were referred to existing smoking cessation hotline services if they requested more comprehensive smoking cessation counseling, only 27 of 1571 participants (1.7%) made such requests, with 7 of 27 participants (25.9%) having confirmed validated abstinence. To enhance the effectiveness of simple interventions, we recommend that health care professionals provide a brief, self-determination theory–based intervention to motivate smokers to quit and then actively refer them to existing smoking cessation services for more intensive counseling. Evidence suggests that active referral of smokers to existing smoking cessation services could effectively increase cessation.^16,36,37^ Finally, in a region with a low prevalence of smoking but many heavy smokers, our results are useful to guide future strategies toward a total ban on tobacco sales in Hong Kong.^38^

### Strengths and Limitations

This trial had some notable strengths. We complied with the Russell standard,^39^ which is the gold standard for smoking cessation trials. According to this standard, biochemical validation of self-reported abstinence must be performed at 6-month and 12-month follow-up. This ensured the consistency of reporting and allowed direct comparisons of these findings with those of other smoking cessation trials.^39^

This trial also had some limitations. First, only 35.9% (1517 of 4228) of eligible smokers consented to participate. Previous smoking cessation trials in outpatient clinics in Hong Kong reported that approximately 70% of smokers had no intention to quit.^17,18,33^ A possible reason for the low participation rate in our trial was that most eligible participants had no interest in quitting and were thus reluctant to participate in a smoking cessation project. The response rate was similar to that of a previous trial on smoking cessation conducted in clinics.^18^ To enhance the generalizability of the findings, we conducted participant recruitment at 4 major acute-care hospitals among different districts of Hong Kong, in which most emergency patients can be approached. Second, our retention rate of 68.5% (1076 of 1571) was similar to that of other smoking cessation trials, which usually had retention rates of approximately 70%.^17,18^ Because such missing participants were analyzed as having no change in baseline smoking status, the quit rates could have been underestimated. A third limitation of this study was that only 50.3% (85 of 169) of self-reported quitters were unreachable or refused biochemical validation. However, the effect based on self-reported quit rates (aRR, 1.32) was smaller than that based on biochemically validated quit rates (aRR, 3.21). Furthermore, the biochemical validation participation rate in the present trial was higher than that in previous trials conducted in outpatient clinics.^17,18,33^ To increase the participation rate, strategies (eg, home or workplace visits) offering incentives and flexible validation schedules could be considered in future studies.

## Conclusions

This brief, low-cost self-determination theory–based intervention for smokers presenting at EDs was effective in increasing the biochemically validated quit rate at 6 months compared with a smoking cessation leaflet. This finding supports the development of evidence-based clinical practice guidelines. If delivered routinely, this simple intervention can offer a cost-effective and sustainable approach to encourage more health care professionals to help smokers quit smoking.

## Notes

Supplement 1. Trial Protocol

Supplement 2. eAppendix. Supplemental Appendix eTable. Sensitivity Analysis for Primary and Secondary Outcomes for Multiple Imputation and Completed Case

Supplement 3. Data Sharing Statement
