# Supplementary material for: Effectiveness of a Brief Self-determination Theory–Based Smoking Cessation Intervention for Smokers at Emergency Departments in Hong Kong: A Randomized Clinical Trial
Source: JAMA Intern Med. 2019 Dec 2;180(2):206–14. doi: 10.1001/jamainternmed.2019.5176 (PMC6902195; doi:10.1001/jamainternmed.2019.5176)
Supplement: Supplement 1. — Trial Protocol [file jamainternmed-180-206-s001.pdf]

Trial Protocol with Statistical Analysis Plan

Section 1: Administrative information

**Title:**	Effectiveness of a Brief, Self-determination Intervention for Smoking Cessation (Immediate or Progressive) Among People Attending Emergency Departments: a Randomised Controlled Trial
**Brief Title:**	Smoking Cessation Among People Attending A&E
**Trial registration:**	ClinicalTrials.gov Identifier: NCT02660957

Section 2: Introduction

Introduction

Cigarette smoking, which causes serious damage to health resulting in many chronic problems including cancer, heart disease, stroke and lung disease [1], is the single most important, preventable cause of death and diseases [2]. It is therefore crucial that healthcare professionals should promote smoking cessation and help patients quit. The Hong Kong government and community have put enormous efforts on raising tobacco tax, smoke-free legislation, law enforcement, health promotion campaigns and smoking cessation services. The prevalence of daily cigarette smokers has been decreasing from 23.3% in 1982 to 11.1% in 2010, which is the lowest in the developed world [3]. Nevertheless, around two-thirds of smokers have never tried to give up smoking [4].

Medical attention at Accident and Emergency Departments (AEDs) of smokers who are in physical discomfort can be developed as an excellent “teachable model” as it provides an invaluable opportunity to initiate smoking cessation. Smokers consulting doctors as an emergency are more liable to change their habits to improve their health. According to the Hospital Authority [5], about 2 million people attend AEDs in Hong Kong each year, of whom 68% are triaged as semi-urgent (level 4) and non-urgent (level 5). The average waiting time for a medical consultation varies among AEDs, but is generally longer than 30 min for triage level 4 and 1-2 h for level 5 [6] – presenting healthcare professionals with a golden opportunity to advise smokers to quit and on the available smoking cessation programmes while they are waiting. However, most of the cessation programmes, including stage-matched interventions, generally take several to more than 30 minutes to implement and hence are not practicable or feasible in busy clinical settings. Indeed, the most common reason cited by healthcare professionals for being unable to help patients to quit smoking is the lack of time, because

they are very busy and cannot spare even a few extra minutes to do so [7]. Other barriers include a lack of training and experience, a lack of confidence in the effectiveness of the interventions, and the absence of incentives and deficiencies in support or requirements by hospital management that these programmes should be implemented. Moreover, our previous smoking cessation projects in outpatient clinics have revealed that many patients are too impatient to undergo a long intervention and some are reluctant to participate for fear that they might miss or experience delays in their medical consultation or other medical procedures. To address such problems, further studies are warranted to develop brief and effective interventions with simple, direct, strong, evidence-based warnings for patients who smoke.

Smoking is addictive and quitting is very difficult, with a high rate of relapse, particularly among those with high nicotine dependency [8, 9]. Our previous smoking cessation projects in outpatient clinics and the community have revealed that many smokers who are reluctant to quit are interested in reducing the number of cigarettes smoked per day. Therefore, another potential option would be to help smokers to reduce the number of cigarettes smoked gradually, with the ultimate goal of complete cessation. Several randomised controlled trials in the West have supported that smoking reduction interventions can help smokers first reduce and eventually quit smoking [10-12].

<u>What we have achieved to date:</u>

We conducted a ‘proof-of-principle’ pilot randomised controlled trial on a very brief (<30 s) smoking cessation intervention with a ‘one in two smokers will be killed by smoking’ warning in medical outpatient clinics in Guangzhou [13]. The results showed that more of the smokers who received this brief advice quit or reduced their smoking than did not. This pilot study suggested that even a very brief intervention on smoking cessation is better than no advice in the ‘real world’ practice for outpatient clinics. Moreover, the study demonstrated the feasibility and acceptability of the implementation of a very brief smoking cessation programme by healthcare professionals in busy clinical settings. In addition, the findings support the need for large randomised controlled trials of brief or minimal interventions with the ‘one in two’ warning

Aims: To test the effectiveness of using a brief, self-determination intervention on smoking cessation (immediate or progressive) for people attending AEDs.

<u>Null hypotheses</u>

<u>Primary</u>

1. There is no difference in the rate of smoking abstinence among subjects who have either received the brief, self-determination intervention on smoking cessation (quit immediately & progressively) or a placebo control intervention at 1 week, and 1, 3, 6, 9, and 12 months.

Secondary

2. There is no difference in the rate of smoking abstinence among subjects who have either received the brief, self-determination intervention on smoking cessation (quit immediately) or a placebo control intervention at 1 week, and 1, 3, 6, 9, and 12 months.

3. There is no difference in the rate of smoking abstinence among subjects who have either received the brief, self-determination intervention on smoking cessation (quit progressively) or a placebo control intervention at 1 week, and 1, 3, 6, 9, and 12 months.

4. There is no difference in the rate of smoking abstinence among subjects who have either received the brief, self-determination intervention on quitting immediately or progressively at 1 week, and 1, 3, 6, 9, and 12 months

Section 3: Study Methods

Study design: A single-blinded multi-centre randomized controlled trial, with two-group pre-test and repeated post-test, between subjects design will be conducted following CONSORT statements.

Settings: The settings will be the AEDs in three hospitals (United Christian Hospital, Queen Mary Hospital and Tuen Mun Hospital). The Nurse-in-Charge and the Chief of Service of the AED of the United Christian Hospital have agreed to participate in the study and provide assistance. We are in the process of inviting another two hospitals to participate in the study, while waiting for the result of the application. All of these hospitals are chosen because they have participated in our previous RCT on cardiac, diabetes and cancer patients and are experienced and cooperative.

Subjects: Chinese patients attending one of the three AEDs at different clusters in Hong Kong (United Christian Hospital, Queen Mary Hospital and Tuen Mun Hospital) for physical discomfort who fulfil the following inclusion criteria will be invited to participate in the study. The inclusion criteria are: (1) aged 18 years or above, (2) triaged as semi-urgent (level 4) or non-urgent (level 5), (3) smoke at least two cigarettes per day and (4) express a willingness to quit smoking. The exclusion criteria are: (1) poor cognitive state or mental illness and (2) participation in other smoking cessation programmes or services. All smoking patients will be referred by triage nurses after determining the

clinical urgency in accordance with the triage guidelines set by the Hospital Authority. Smoking patients will then be approached by our research assistants.

The conceptual framework: The intervention is guided by the social cognitive theory and the self-determination theory. According to social cognitive theory, self-efficacy is an important personal determinant of human behaviour and has been defined as the belief in one's capability to engage in behaviour to solve difficult tasks. This belief influences decisions on whether a certain form of behaviour will be adopted and maintained [16]. Because self-efficacy is built on a successful experience of overcoming challenging tasks [16], smokers who have more successful experiences in reducing cigarette consumption tend to have higher levels of self-efficacy. Some evidence has shown that a reduction in smoking may lead to greater self-efficacy to resist smoking, which could increase subsequent quitting [17].

According to the self-determination theory [18], behavioural regulation is more autonomous when it is internalized, as opposed to being regulated by external factors (e.g., orders from family members, friends, or healthcare professionals). Compared with external regulation, autonomous regulation is associated with increased self-efficacy, greater behavioural persistence, long-term behavioural changes, and more positive health behaviour [16]. Autonomy is another influential determinant of behaviour which is emphasized by freedom of choice [18]. Studies have shown that patients who have an opportunity to decide on their own treatment may feel more eager to comply with instructions [19]. The subjects in this study will be allowed to select their own schedules of quitting after discussions with the trained research assistant, such as to quit immediately or to reduce the number of cigarettes smoked progressively with the ultimate goal of complete cessation over an acceptable period. It is anticipated that the subjects will show more willingness to adhere to their own schedule as a result of an increase in autonomy. Moreover, some evidence has shown that autonomy is positively associated with competence [20]; that is, people who have greater autonomy demonstrate higher competence in achieving behavioural change. Consequently, autonomy will facilitate their gradual cessation of smoking.

Randomization: The method of simple complete randomization will be adopted. Subjects will be randomly allocated into one of the two groups: A placebo control group or the intervention group. Randomization will be performed by opening of a serially labelled, opaque and sealed envelope (SNOSE) with a card inside indicating the randomly allocated group by a research assistant (independent to those who provide interventions to subjects). The random numbers for group

assignment will be generated by another research assistant of the project using a personal computer before subject recruitment. Allocation concealment will be ensured. With random assignment of individuals to the intervention or control groups, the possibility existed for interaction between the two groups of subjects while waiting for medical consultation. A precautionary measure will be taken in the AEDs by assigning the same waiting area or cubicle to either the control or the intervention subjects.

Intervention: Subjects in the intervention group will be allowed to select their own schedules of quitting after discussing their situation with the trained research assistant (quit immediately (QI), or quit progressively (QP) with the ultimate goal of completing cessation over an acceptable period). Subjects in the intervention QI subgroup will receive a smoking cessation leaflet plus a brief intervention using the AWARD model: (a) Ask about smoking history, (b) Warn about the high risk, (c) Advise to quit now, as quitting can greatly reduce risks, (d) Refer smokers to a smoking cessation clinic or to smoking cessation hotline: 1833183, and (e) Do it again: repeat the intervention and encourage smokers who fail to quit or relapse to try again during each telephone follow-up. For the warning message, the trained research assistant will repeat the following in a standardized manner: (draft, will be improved after pilot testing) 'The World Health Organization says that based on medical research, one out of two smokers will be killed by smoking. This 50% risk is very high. You have decided to reduce smoking as you know this is good for you.' The whole intervention will be limited to less than 1 min or slightly longer if necessary.

Subjects in the QP subgroup will receive a smoking cessation leaflet plus brief intervention using AWARD model similar to the QI subgroup, except for the 'advice' being given to them. Subjects will be asked to think about a tailored quitting schedule for themselves after the discussions with the trained research assistant. The trained research assistance will motivate the subjects to quit progressively over an agreed period not to exceed 6 months.

Rationale for using a brief intervention programme

A brief intervention with a small to moderate effect size can potentially benefit a large number of smokers and increase smoking cessation within the community if it is carried out routinely in clinical practice by all or most healthcare professionals or people with minimal training. It is also the most cost-effective smoking cessation programme, because no extra or minimal funding is needed to provide the venue, manpower, and other expenses (but incentives or payments to healthcare

professionals and follow-up support would be needed). There is no evidence that longer interventions are more effective than shorter interventions [14]. Brief cessation interventions have been shown to be effective with strong evidence from our randomised controlled trials [13] and in systematic reviews [14, 15].

Follow-up intervention

For the subsequent telephone follow-up in the intervention group, information on reduction and cessation will be collected, followed by a 'booster' intervention, which will repeat the health warning that 'one in two smokers will be killed by smoking', positively encourage them to reinforce their efforts, and remind subjects in the QP subgroup of their next reduction target. For example, if subjects report that they have reduced or stopped smoking, the trained research assistant will say: 'Congratulations on your successful reduction/abstinence. How confident are you that you will be able to keep on quitting according to your plan (for QI). How much do you plan to reduce further and how would you plan to smoke increasingly less (for QP)? We are confident that you can succeed in quitting and lead a healthier life.' However, if subjects report that they failed to quit or have not reduced smoking, the trained research assistant will say: 'Don't be disappointed. Would you please tell me how you would try and plan to quit or reduce smoking now or in the near future?' The trained research assistant will give brief suggestions to the subjects based on their responses and reinforce the message that quitting smoking is good for their health and that they can do it.

Six consecutive (1 week, and 1, 3, 6, 9, and 12 months) follow-ups will be conducted over the telephone by trained interviewers. Outcome assessment will first be carried out with blinding to the group assignment. Then, the group status will be disclosed so that the intervention group will receive the booster intervention and the placebo control group will not. We propose to extend the follow-up period from 12 months to 24 months for future study if other funding becomes available.

Subjects in the placebo control group will receive a smoking cessation leaflet published by the Hong Kong Council on Smoking and Health (COSH), as will the intervention group. Moreover, subjects in the placebo control group will undergo a similar schedule of telephone follow-up as those in the intervention group. They will receive a 'placebo' intervention with a 'placebo booster' of the same duration on increasing physical activity and fruit and vegetable intake.

Instruments: A structured questionnaire will be developed by adopting or modifying international and/or locally validated instruments. The questionnaire gathers information including smoking and quitting history, stage of readiness to quit, and demographic information such as age, gender, and marital status. The demographic and clinical information will be obtained from the AEDs medical records.

Outcome measures

Baseline data, including demographics, health status, and smoking history, will be obtained from each patient using a structured questionnaire, administered in face-to-face interviews by the trained research assistant prior to randomisation.

The primary outcome measure is self-reported 7-day point prevalence of abstinence at 1 week, and 1, 3, 6, 9, and 12 months. Secondary outcomes are: (i) biochemically validated abstinence at 6 and 12 months, (ii) self-reported reduction of ≥ 50% in cigarette consumption at 1 week, and 1, 3, 6, 9, and 12 months, and (iii) b biochemically validated reduction of > 50% in cigarette consumption at 6 and 12 months. Patients who have successfully quit smoking or reduced cigarette consumption by at least 50% at 6-, and 12 month will be invited to biochemical validation tests. We will offer HK$300 per client to cover their travel expenses and time cost. From our previous experience, such an incentive is necessary to secure a sufficiently high response rate. It is a common practice in clinical trials to include financial incentives in order to encourage subjects to come back for biochemical validation and to sponsor their travelling expenses at follow up. Note that the incentive is not for joining the trial. The biochemically validated 7-day point prevalence of abstinence will be confirmed by a carbon monoxide level in expired air < 9 parts per million (p.p.m.) and saliva cotinine level < 115 ng/ml in parallel test [21]. The biochemically validated reduction of > 50% in cigarette consumption will be confirmed by exhaled carbon monoxide. Subjects will be considered non-reducers if they have reduced their exhaled carbon monoxide level by < 1 ppm compared with baseline. These biochemical validation methods have been used in previous smoking cessation and reduction studies [8, 9].

Sample size calculation:

Sample size calculation is based on the main outcome variable according to the main hypotheses, the 7-day point prevalence self-reported quit rate at 6-month in the intervention group is higher than that

in the control arm of the RCT. With reference to our previous RCT testing the effect of smoking reduction for smokers not willing to quit smoking [8], we assume that the quit rate was 10.2% in the control group and 17.0% in the intervention group. With $\alpha = 0.05$, $\beta = 0.2$ (power = 80%), two-tailed z test, the sample size is estimated to be 796 in total. Taking into account the retention rate of about 90% from the previous RCT [8], we plan to recruit at least 886 subjects (443 X 2 arms) for the current RCT.

With reference to the Hospital Authority Statistical Report [5], there are overall 549,856 attendances to the AEDs of United Christian Hospital, Queen Mary Hospital and Tuen Mun Hospital during 2011 to 2012. According to our previous smoking cessation projects conducted in out-patient clinics, about 5% patients were smokers. However, more than 50% of these smokers were reluctant to quit. Therefore, in a rough estimation, there will be around 5,000 eligible subjects in the three selected AEDs each year. Therefore, we have confidence that we can recruit adequate subjects (886) according to the power analysis within a 9-month (39-week) recruitment period. Data collection will be performed at the AEDs from 9am to 5pm on the weekdays.

We will offer HK$100 to each eligible subject who participate in the study and complete six consecutive (1 week, and 1, 3, 6, 9, and 12 months) telephone follow-ups. According to our experience with an on-going RCT on testing the effectiveness of an intervention of smoking fathers living with non-smoking spouse and infant, monetary incentive of $100 can effectively boost the participation rate from about 30% to 60% at 6-month follow-up.

Training and quality assurance of the research assistants: All the research assistants will be provided with specific training workshop by the PI and Co-Is prior to the commencement of the study. They will be equipped with the necessary knowledge and skills to deliver both the smoking cessation advice using the AWARD model. Regular case conference, quality checks through audio-taping, and audit procedures will be conducted to ensure and maintain the quality and uniformity of the interventions. Moreover, the research assistant(s) will be trained to screen eligible case, conduct baseline and follow-up surveys and procedures of biochemical validations (saliva cotinine test and exhale CO test) by PI and Co-Is. Briefing sessions will also be provided to AEDs nurses.

Since the same batch of AEDs nurses working in the setting would contact subjects in both the intervention and control group, a quality assurance mechanism will be used to prevent the contamination of intervention and control group. Apart from assigning the same waiting area or

cubicle to either the control or the intervention subjects (mentioned in point 5: Randomization), a clear instruction and reminder will be given to the participating nurses before the start of the RCT study. Moreover, we will explain the principle of RCT and reinforce them the different treatments for the intervention and control groups. Additionally, doctors and nurses will be reminded that their care will be as usual.

Quality and data security control: This study will follow the protocol of quality assurance developed by a previous project [22]. The PI/ co-Is, project coordinator and research assistants will set up orientation meetings with COS, nurse managers and frontier nurses to explain the protocol, the flow of logistics and examine the physical facilities available in the hospital. The PI/ co-Is will answer any queries raised by doctors and nurses immediately at any time point. The project coordinator will be present during the first week to monitor the subject recruitment and data collection process. All blank and completed instruments will be sealed in separate opaque envelopes, which will be kept in a locker with keys provided by hospitals, while the project coordinator will collect the filled instruments weekly by hand. All collected instruments will be saved in a locker with keys in our School. The project coordinator and other research assistant(s) of this project will be responsible to input the data into SPSS, with the dataset encrypted in an assigned PC in our School. Only the PI, co-Is, project coordinator, relevant research assistants, the independent data monitoring committee (IDMC), and the Institution of Review Board (IRB) may have rights to access the data (in hard or soft copy).

Section 4: Statistical principle and analysis

Statistical software: The Statistical Package for Social Sciences (SPSS Version 23.0, SPSS Inc., Chicago, IL) software for Windows was used for analysis of the quantitative data.

Analysis methods: Data analysis will be performed using the Statistical Package for Social Science. We will compare the baseline characteristics of the patients by χ2 test for categorical variables and F-test for continuous variables between the intervention and the control group. We shall use the χ2 test to assess the effect of intervention and calculate the crude odds ratio (OR) with 95% confidence interval (CI) for the primary and secondary outcomes. Those who are lost to follow-up at 1 week, and 1, 3, 6, 9, and 12 months, or refuse to participate in the validation tests, will be treated as smokers with no reduction in cigarette consumption compared with (a)

baseline, as the main analysis (by intention to treat), (b) the most recent level) and (c) complete case (per protocol) analysis by excluding subjects with missing data as a sensitivity analysis.

Independent data monitoring committee (IDMC): An IDMC will be set up with local and international experts (3-5 people) in smoking cessation trials to review the progress every 6 months to advise and set guidelines for continuing or stopping of subject recruitment as necessary.

Timetable of work: The study is expected to be completed in 24 months.

Existing facilities: Essential facilities such as office space, computer, statistical packages, software, printer and telephone sets will be provided by the School of Nursing, the University of Hong Kong.

Purpose of potential for implementation of the results

It is crucial for healthcare professionals to help patients quit smoking. But most existing smoking cessation interventions generally take several to more than 30 minutes to implement and hence are not feasible in busy clinical settings. Further studies are warranted to develop brief and effective interventions with simple, direct, strong, evidence-based warnings for patients who smoke. Strong evidence from a randomised controlled trial on the effectiveness of brief and practicable interventions would have the greatest potential to ensure that interventions would be carried out routinely in clinical and community settings, benefiting a large number of smokers. We shall conduct an RCT on a brief, self-determination intervention for smoking cessation (immediate or progressive) among people attending emergency departments. To our knowledge, this is the first study on a brief, self-determination intervention on smoking cessation for people attending emergency departments, which will generate new knowledge and evidence, with major clinical and public health implications. Specifically, this innovative, cheap, and brief intervention to achieve a greater level of smoking abstinence will make an important contribution to the evidence-based practice of brief smoking reduction programmes. If proven to be effective, and even if the effect size of the brief intervention is smaller than more intensive brief interventions, it should be more cost-effective and sustainable in its ability to improve the quality of life of more smokers, and thus might save more lives. The results should support the development of

clinical practice guidelines. Most importantly, the results will motivate more healthcare professionals and others (including family members of smokers) to help smokers routinely in clinical and community settings, prompting other researchers to design and evaluate brief smoking cessation programmes.

Reference:

1. U.S. Department of Health and Human Services. *How Tobacco Smoke Causes Disease: The Biology and Behavioral Basis for Smoking-Attributable Disease: A Report of the Surgeon General*. Atlanta, GA: U.S. Department of Health and Human Services, Centers for Disease Control and Prevention, National Center for Chronic Disease Prevention and Health Promotion, Office on Smoking and Health, 2010.
2. World Health Organization. *Report on the Global Tobacco Epidemic: Enforcing bans on tobacco advertising, promotion and sponsorship.* Switzerland, Geneva: World Health Organization; 2013.
3. Census & Statistics Department. *Pattern of Smoking. Thematic household survey (report no. 48)*. Hong Kong: Hong Kong Census & Statistics Department; 2011.
4. Census and Statistics Department, Social Surveys Section. *Thematic Household Survey Report No. 36: Pattern of smoking.* Hong Kong: Census and Statistics Department, 2008.
5. Hospotal Authority. Hospital Authority Statistical Report (2011-2012). http://www.ha.org.hk/upload/publication_15/471.pdf.
6. Hospital Authority (2010). Management of Accident & Emergency Services. Internal audit report. Hong Kong: Hospital Authority.
7. Vogt F, Hall S, Marteau TM. General practitioners' and family physicians' negative beliefs and attitudes towards discussing smoking cessation with patients: a systematic review. *Addiction* 2005; **100**: 1423–31.
8. Chan SSC, Leung DYP, Abdullah ASM, *et al*. A randomized controlled trial of a smoking reduction plus nicotine replacement therapy intervention for smokers not willing to quit smoking. *Addiction* 2011; **106**: 1155-1163.
9. Wennike P, Danielsson T, Landfeldt B, *et al*. Smoking reduction promotes smoking cessation: results from a double blind, randomized, placebo-controlled trial of nicotine gum with 2-year follow-up. *Addiction* 2003; **98**: 1395-1402.
10. Batra A, Klingler K, Landfeldt B, *et al*. Smoking reduction treatment with 4-mg nicotine gum: A double-blind, randomized, placebo-controlled study[ast]. *Clin Pharmacol Ther* 2005; **78**: 689-696.
11. Carpenter MJ, Hughes JR, Solomon LJ, *et al*. Both smoking reduction with nicotine replacement therapy and motivational advice increase future cessation among smoker unmotivated to quit. *J Consult Clin Psychol* 2004; **72**: 371-381.
12. Shiffman S, Ferguson SG, Strahs KR. Quitting by Gradual Smoking Reduction Using Nicotine Gum: A Randomized Controlled Trial. *Am J Prev Med* 2009; **36**: 96-104.e101.

13. Lin PR, Zhao ZW, Cheng KK, *et al*. The effect of physician's 30 s smoking cessation intervention for male medical outpatients: a pilot randomized controlled trial. J Public Health 2013; **35**: 375–383.
14. Aveyard P, Foulds J. *The 2008 US Clinical Practice Guideline: the key recommendations and a commentary*. 2009. www.treatobacco.net.
15. Stead LF, Bergson G, Lancaster T. Physician advice for smoking cessation. Cochrane Database Syst Rev 2008; 16: CD000165.
16. Bandura A. *Self-efficacy: The exercise of control*. New York: Freeman, 1997.
17. Broms U. Korhonen T, Kaprio J. Smoking reduction predicts cessation: longitudinal evidence from the Finnish adult twin cohort. *Nicotine Tob Res* 2008; **10**: 423-428.
18. Deci E, Ryan R. *Handbook of self-determination research*. Rochester, NY: University of Rochester Press, 2002.
19. Williams GC, Cox EM, Kouides R, *et al*. Presenting the facts about smoking to adolescents: effects of an autonomy-supportive style. *Arch Pediatr Adolesc Med* 1999; **153**: 959-964.
20. Williams GC, McGregor HA, Zeldman A, *et al*. Testing a self-determination theory process model for promoting glycemic control through diabetes self-management. *Health Psychology* 2004; **23**: 58-66.
21. Jarvis MI, Tunstall-Pedoe H, Feyerabend C, *et al*. Comparison of tests used to distinguish smokers from non-smokers. *Am J Public Health* 1987; **77**: 1435-1438.
22. Chan SSC, Lam TH, Lau CP. A randomized controlled trial of a nurse delivered staged-matched smoking cessation intervention to promote heart health of cardiac patients (HKU7224/01M). Final report submitted to the Research Grants Council, 2007.
