# Supplementary material for: Effectiveness of a Brief Self-determination Theory–Based Smoking Cessation Intervention for Smokers at Emergency Departments in Hong Kong: A Randomized Clinical Trial
Source: JAMA Intern Med. 2019 Dec 2;180(2):206–14. doi: 10.1001/jamainternmed.2019.5176 (PMC6902195; doi:10.1001/jamainternmed.2019.5176)
Supplement: Supplement 2. — eAppendix. Supplemental Appendix eTable. Sensitivity Analysis for Primary and Secondary Outcomes for Multiple Imputation and Completed Case [file jamainternmed-180-206-s002.pdf]

Supplementary Online Content

Li WHC, Ho KY, Wang MP, et al. Effectiveness of a brief self-determination theory–based smoking cessation intervention for smokers at emergency departments in Hong Kong: a randomized clinical trial. *JAMA Intern Med*. Published online December 2, 2019. doi:10.1001/jamainternmed.2019.5176

eAppendix. Supplemental Appendix

eTable. Sensitivity Analysis for Primary and Secondary Outcomes for Multiple Imputation and Completed Case

This supplementary material has been provided by the authors to give readers additional information about their work.

© 2019 Li et al. *JAMA Internal Medicine.*

eAppendix. Supplemental Appendix

Interventions

Intervention group

After discussing their situation with our trained counsellor, intervention group participants were allowed to choose their own quit schedule: quit immediately (QI), or quit progressively (QP) with the ultimate goal of complete cessation at 6 month

QI subgroup

Participants received a smoking cessation leaflet published by the Hong Kong Council on Smoking and Health plus a series of brief interventions using the AWARD model. The nurse counsellor (i) asked about smoking history (ASK), (ii) warned subjects about their increased risk of premature death (Warn), (iii) advised participants to quit immediately (Advice), (iv) referred participants to existing cessation services by giving them a hotline number (Refer) and (v) do it again when participants failed to quit (DO-it-again). The counsellor provided a standardised warning message: 'The World Health Organization warns that one out of two smokers will be killed by smoking. Recent medical research has shown that for those who started smoking at a young age, two out of three will die from smoking. This 1/2 to 2/3 risk is very high and dangerous. You have decided to quit smoking as you know this is good for you.' The whole intervention took about 1 minute.

© 2019 Li et al. *JAMA Internal Medicine.*

QP subgroup

Participants received a smoking reduction leaflet developed by the present authors, which contained reduction strategies and a suggested plan to reduce smoking (reduce cigarette consumption by 15% in the first week, 30% in the first month and 50% in the third month, and eventually quit smoking in the 6th month). In addition, participants received a series of brief interventions using the AWARD model. These were similar to the QI subgroup, but differed in the advice given. The counsellor motivated participants to reduce their cigarette consumption according to the suggested plan or think about a tailored quitting and quit at their own pace but noted that the whole process should not exceed 6 months.

Follow-up intervention

Four consecutive telephone follow-ups (1, 3, 6 and 12 months) were conducted by trained counsellors. The counsellors first conducted outcome assessments with blinding as to group allocation. Then, the group status was disclosed so that the intervention group received a booster intervention while the control group did not.

The counsellor repeated the standardised warning regarding mortality risk (as described above) and strongly encouraged participants to reinforce their efforts. They also reminded those in the QP subgroup of their next reduction target. The counsellor emphasised and congratulated participants if they had reduced/stopped smoking or had not relapsed. For example, the

counsellor would say: ‘Congratulations on your successful reduction/abstinence. How confident are you that you will be able to keep on quitting according to your plan?’ (for QI) or ‘How much do you plan to reduce further and how would you plan to smoke increasingly less?’ (for QP) ‘We are confident that you can succeed in quitting and lead a healthier life.’

If participants reported that they had relapsed, failed to quit or not reduced smoking, the counsellor would say: ‘Don’t be disappointed. Would you please tell me how you would try and plan to quit or reduce smoking now or in the near future?’ The counsellor also offered brief suggestions based on participants’ responses and reinforced the message that quitting smoking was good for their health, and that they could succeed. Each booster intervention usually took about 2 minutes.

Training and quality assurance

All counsellors were retired nurses who had attended a specific training workshop conducted by the research team before the study started to ensure that they were equipped with the necessary knowledge and skills to deliver smoking cessation advice using the AWARD model. Regular case conferences, quality checks through audio-taping and audit procedures were conducted to maintain the quality and uniformity of the interventions. The counsellors were also trained to screen for eligible subjects and conduct baseline/follow-up surveys and biochemical validation (saliva cotinine test and exhale CO test).

© 2019 Li et al. *JAMA Internal Medicine.*

Control group

Participants in the control group received a smoking cessation leaflet published by the Hong Kong Council on Smoking and Health, and schedule of telephone follow-ups similar to the intervention group. They received a placebo intervention and placebo boosters of the same duration, promoting more physical activity and fruit and vegetable intake.

All subjects who participated in the trial and completed six consecutive (1 week, and 1, 3, 6, 9, and 12 months) telephone follow-ups received HK$100 (equivalent to US$13) and those who participated in the biochemical validation test at 6, and 12 months received HK$300 (equivalent to US$39) to cover their travel expenses and time cost.

The cost of interventions

The operating costs (USD) related to the training ($411), recruitment and intervention delivery ($27,786), intervention materials ($366) and brief telephone booster intervention ($246) amounted to $28,809. The mean costs for delivering the brief smoking cessation intervention using the AWARD model and minimal general advice (physical activity and fruit and vegetable) intake were $18.570 and $18.105, respectively. The differences in costs were mainly attributable to participants receiving different materials (smoking cessation warning leaflet, smoking reduction leaflet and self-help booklet).

© 2019 Li et al. *JAMA Internal Medicine.*

eTable. Sensitivity Analysis for Primary and Secondary Outcomes for Multiple Imputation and Completed Case

	Intention-to-treat [a] Adjusted RR (95% CI)[c]	P value	Completed case [b] Adjusted RR (95% CI) [c]	P value
6 month				
Biochemically validated abstinence, (Primary outcome)	3.21(1.74, 5.93)	<.001	3.01(1.66, 5.46)	.02
Self-reported 7-day point prevalence of abstinence	1.32(0.90, 1.95)	.11	1.24(0.48, 3.20)	.25
12 month				
Biochemically validated abstinence, (Primary outcome)	2.23(1.25, 3.97)	.004	1.95(1.03, 3.72)	.02
Self-reported 7-day point prevalence of abstinence	1.46(1.06, 2.19)	.04	1.63(1.02, 2.74)	.04

© 2019 Li et al. *JAMA Internal Medicine.*
