# Supplementary material for: Effectiveness of a Brief Self-determination Theory–Based Smoking Cessation Intervention for Smokers at Emergency Departments in Hong Kong: A Randomized Clinical Trial
Source: JAMA Intern Med. 2019 Dec 2;180(2):206–14. doi: 10.1001/jamainternmed.2019.5176 (PMC6902195; doi:10.1001/jamainternmed.2019.5176)
Supplement: Supplement 3. — Data Sharing Statement [file jamainternmed-180-206-s003.pdf]

Data Sharing Statement

Li. Effectiveness of a Brief Self-determination Theory-Based Smoking Cessation Intervention for Smokers at Emergency Departments in Hong Kong. *JAMA Intern Med*. Published December 02, 2019. 10.1001/jamainternmed.2019.5176

Data
Data available: No

Additional Information
Explanation for why data not available: All data are belong to the funder (Food and Health Bureau, Hong Kong SAR). The data can be accessed only with the permission by the Bureau
